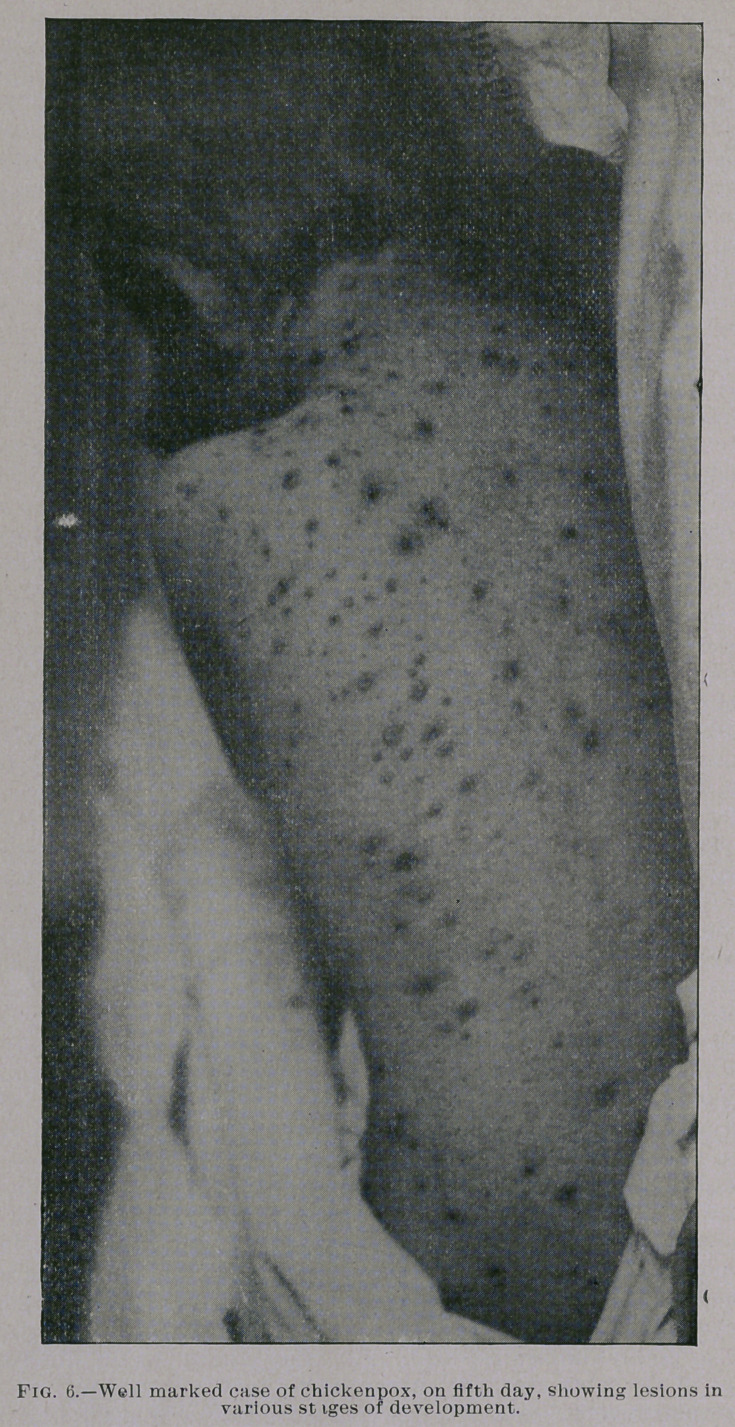# Remarks on the Present Mild Type of Smallpox; the Symptoms and Diagnosis*Reprinted from *The Philadelphia Medical Journal*.

**Published:** 1901-02

**Authors:** William M. Welch

**Affiliations:** Philadelphia


### Remarks on the Present Mild Type of Smallpox; the Symptoms and Diagnosis.*

BY WILLIAM M. WELCH, M. D., OF PHILADELPHIA.

Two or three years ago, smallpox of an unusually mild type appeared in the Southern States, and the disease, from a diagnostic standpoint, was variously regarded by the physicians. Some looked upon it as chicken pox, others called it impetigo contagiosa, a few thought it was a cutaneous affection of some new and strange variety, while a considerable number believed it to be smallpox. I have been informed that the profession was quite equally divided on the question of smallpox or chickenpox, about as many calling it the one as the other. The disease was recognized as infectious, as it was seen to spread from one person to another, and from town to town, until the epidemic was wide-spread and the cases numerous. But wherever the disease was seen it was of the same mild type, and rarely resulted in death. The strange thing about it was, apart from its mildness, that it seemed to attack negroes in preference to white people. Those who took the disease were, as a rule, confined to the house only during the initial stage. After the appearance of the eruption the patients would frequently go about their work as usual, feeling but little, if any, indisposed. Not infrequently they were employed gathering cotton and preparing it for market while the eruption was developing or the scabs falling off, and it is believed that the infection was spread to distant localities by this article of commerce. I saw a case of smallpox in this city last year in which the infection was believed to have been received from a bale of cotton brought from the South. The case occurred in a man employed in a mill where cotton goods were manufactured, and at a time when no cases of smallpox had been

---

*Reprinted from *The Philadelphia Medical Journal.*

seen in the city for a period of between two and three years. The man had not been out of the city for a long time, and was constantly at his work. In seeking for the source of the infection in this case, I could arrive at no other conclusion than that it had been derived from a bale of cotton. This man, after falling ill, gave the disease to his brother, who roomed with him, and these two were the only cases of smallpox in Philadelphia until several months subsequently. The type in both cases was mild, though the disease was well pronounced. Recovery, of course, followed.

It is said that the disease was transmitted into the Southern States from Cuba, where it prevailed during the Spanish-Cuban war. In explanation of the mild type of the affection, it has been suggested that smallpox in the tropics is less severe than in a cold climate. I am not sure that this is true, but even if it is, I see no reason why the disease should not assume its old and familiar form when the infection is conveyed to the Middle and Northern States; but up to the present time it has shown no such tendency. What it will do when cold weather sets in remains to be seen.

From the South the disease spread to very many of the Northern States and it was everywhere so mild and frequently so atypical that the same difficulty in the matter of diagnosis, as already referred to, was experienced by the physicians of these States. In several counties of our own State, as, for instance, Bedford, Somerset, Allegheny, and Philadelphia, the cases were so numerous as to almost constitute an epidemic. In all of these localities I feel sure that the earlier cases were not recognized, and the affected persons were permitted to roam about at pleasure. The diagnosis of chickenpox was, perhaps, the most common error made by physicians wherever the disease occurred. It was not uncommon in this city for patients to apply at dispensaries for treatment, take their position in the waiting-room, and, after an examination by a physician, be provided with a salve with instructions to apply it to the local lesions. I do not know what the diagnosis was in such cases, but the disorder was evidently regarded as some form of skin disease. Next to chickenpox, the most common error of diagnosis was, I think, impetigo contagiosa. According to Drs. Lee and Atkinson, this diagnosis was stoutly maintained by some of the physicians of Bedford, Pa. "Cuban itch" is another name which I am informed was given to the disorder. Some of the colored people who were treated in the municipal hospital called it "elephant's itch." If it were really itch this would seem to be, in view of the size of the vesicles, a very appropriate name. These colored people alleged that the disease originated at Norfolk, Va., by some of their people sleeping on straw on which elephants had slept. But the most popular name for the disorder among the colored people was "the bumps."

Considering the rare opportunity of late years for physicians to study smallpox clinically, and the unprecedented mildness of the disease at the present time, I am not surprised at the frequent errors of diagnosis. We know that the young physician begins his lifework with no other knowledge of smallpox than that which he has derived from books or the didactic lecture. The college is careful to provide for him almost every possible variety of clinical instructions on diseases that are not contagious, but on the contagious variety he receives absolutely none. This is not the fault of the colleges nor of the students, but boards of health or civic authorities which control the hospitals in which contagious and infections diseases are treated. If such hospitals were open for clinical instruction, it would be impossible to estimate the benefit therefrom to the student primarily, and secondarily to the public. It is here that smallpox may be seen at once in its various stages,

FIG. 1.—Mild form of smallpox in an unvaccinated adult, on the fifth day of eruption.

[This and the following illustrations are from photographs taken by Dr. Jay F. Schamberg.]

and in every possible type. The didactic lecturer, as a rule, treats only of typical cases, but in the hospital both typical and atypical cases may be studied. If, therefore, clinical instruction was so comprehensive as to include all contagious and infectious diseases, mistakes of diagnosis would be less frequent, and, in an outbreak

FIG. 2.—Mild form of smallpox in an unvaccinated child, on seventh day of eruption.

of smallpox, the earlier as well as the atypical cases would more readily be recognized, and thus widespread and fatal epidemics might more frequently be prevented.

In an experience of twenty-nine years of hospital work, which includes a study of over 5,500 cases of smallpox, I must say I have never seen cases present, uniformly, so mild a type as during the present year, nor have I been able to find in the vast amount of literature published on the subject any account of a similarly mild epidemic in this or any other country. It is true that not all cases are equally mild. You will notice from the photographs which I show you, taken by my friend Dr. Jay F. Schamberg, that the eruption was quite thickly set in some of the cases—so thickly as to show a considerable degree of confluence on some parts of the body, particularly the face, while in others the eruption was very sparsely seen. It was only the best marked cases that were selected for photographing. Indeed, in some of the mildest cases, it was impossible to count as many as a dozen pustules, even on persons who had never been vaccinated. The vast majority of the patients would not remain in bed after the eruption appeared. They would dress up in their clothing, walk about and indulge in various pranks, tricks and games. It was a novel sight to me to see smallpox patients, negroes, unvaccinated, at about the eighth or tenth day of the eruption, engaging in a game of baseball. I have not seen more than two or three cases during the present prevalence of the disease which showed symptoms at all serious.

The number of patients who have come under my observation in the hospital during the present year is 128, without a single death occurring. Of this number 110 were unvaccinated and 17 were vaccinated in infancy, and one after exposure to the infection. Six were white and 122 black; 92 were male and 36 female. The quality of the vaccine marks of those who were vaccinated and the ages of all the patients may be seen in the following tables:

TABLE 1.

| | Cases. | Deaths. |
|---|---|---|
| Vaccinated in infancy (good scar) | 5 | 0 |
| " " " (fair scar) | 2 | 0 |
| " " " (poor scar) | 10 | 0 |
| Postvaccinal cases | 17 | 0 |
| Unvaccinated cases | 110 | 0 |
| Vaccinated after exposure | 1 | 0 |
| Total | 128 | 0 |

TABLE II.

| Ages. | Cases. | Deaths. |
|---|---|---|
| Under 1 year | 1 | 0 |
| 1 to 5 years | 7 | 0 |
| 5 to 10 years | 4 | 0 |
| 10 to 15 years | 3 | 0 |
| 15 to 25 years | 58 | 0 |
| 25 years and upwards | 55 | 0 |
| Total | 128 | 0 |

The seventeen cases of smallpox which occurred after vaccination are included in the age periods of the above table as follows: Ten to fifteen years, one; fifteen to twenty-five years, eight; twenty-five years and upwards, eight. These patients had all reached the age when the prophylactic effect of the infantile vaccination is frequently found to be diminished or absent. Of the total number of patients the vast majority was over fifteen years of age and unvaccinated. They were nearly all negroes who had been born and raised in the South, mostly in Virginia. I have noticed that for some reason or other, vaccination is greatly neglected in the Southern States, particularly among the negroes. The prophylactic power of vaccination is clearly evident from the fact that so few of the cases of smallpox occurred in persons who were vaccinated. Besides, it is believed that but for vaccination the disease would have become widespread and assumed an epidemic form of immense proportion, since so many of the persons affected were not ill enough to be confined to the house, but, on the contrary, mingled quite freely with the public by visiting dispensaries, riding on trolley and steam cars, walking and driving on the streets, and the like.

Previous to the present year, the last time that smallpox appeared in this city and spread to any considerable extent was in the years 1894-95. At that time the disease presented its usual clinical phenomena throughout its various stages, and was in many cases very severe, although not so generally so as in preceding epidemics. This is evident from the mortality rate, which was only 18% in the unvaccinated, as against 58.38%, which was the average death rate in the hospital of all previous epidemics as far back as 1870. In the extremely malignant epidemic of 1871-72, the death rate in the unvaccinated cases was as high as 64.41%. While the death rate of 18% was very low in comparison with my previous experience, it is not, however, unprecedentedly low, as has been the case almost everywhere in this country during the present prevalence of smallpox. Even before vaccination was discovered small outbreaks of the disease were occasionally met with in certain localities in which the mortality was not above 18%, while the average death rate from natural smallpox during the eighteenth century was, according to available statistics, not less than 40%.

In order that the results seen during the present year may be contrasted with my previous experience, I will present the following tables:

TABLE III—SHOWING THE STATISTICAL DETAILS OF THE SMALLPOX CASES TREATED IN THE MUNICIPAL HOSPITAL IN 1894-95.

| | Cases. | Died. | Percent. |
|---|---|---|---|
| Vaccinated in infancy (good scars) | 57 | 0 | 0 |
| " " " (fair scars) | 34 | 3 | 8.82 |
| " " " (poor scars) | 32 | 6 | 18.75 |
| Total number vaccinated | 123 | 9 | 7.31 |
| Unvaccinated cases | 156 | 9 | 17.95 |
| Vaccinated after exposure | 24 | 2 | 8.33 |
| Total | 303 | 38 | 12.87 |

TABLE IV.—SHOWING STATISTICAL DETAILS OF THE SMALLPOX CASES TREATED IN THE MUNICIPAL HOSPITAL FROM 1870 UNTIL 1890..

| | Cases. | Died. | Percent |
|---|---|---|---|
| Vaccinated in infancy (good scars)........... | 1,412 | 124 | 8.78 |
| " " " (fair scars)........... | 666 | 98 | 14.71 |
| " " " (poor scars)........... | 1070 | 290 | 27.10 |
| Postvaccinal cases.............................. | 3,148 | 512 | 16.26 |
| Unvaccinated cases................................ | 1,759 | 1,027 | 58.38 |
| Unclassified cases.................................. | 93 | 23 | 24.73 |
| Total ................................................ | 5,000 | 1,562 | 31.24 |

The onset of the present mild type of smallpox does not differ greatly, except in degree, from that commonly seen in the severer forms of the disease. The patient is usually taken suddenly ill. A chill, more or less marked, is commonly an early symptom. It may be so mild as to constitute only a slight rigor, so slight, indeed, as to pass quite unnoticed by the patient. This is followed by the usual evidences of pyrexia. The temperature may vary from 101° F. to 105° F. High temperature is apt to be accompanied by great restlessness. At the same time irritability of the stomach occurs, which may be only slight, but is often intense and distressing, and may continue throughout the entire stage of the initial fever. Lumbar pain is also very common as an early symptom, and this, too, may be slight or severe. Sometimes it is absent altogether. Encephalic symptoms very frequently accompany this stage. In adults, headache is often severe, and when the temperature is high there may be delirium. In children there is apt to be somnolency, and convulsions often occur. The tendency to syncope, the marked dizziness on assuming the erect position, and the excessive prostration, so common in severe cases of smallpox, are often quite absent in the present mild type of the disease. Indeed, according to information obtained from many of the patients who came under my notice, the entire initial stage was so mild that they were not obliged to remain constntly in bed; some even stated that they had scarcely been ill at all, and yet on close interrogation I was able to learn that all had suffered to some degree from nearly all the symptoms I have described. In a few the initial stage was marked by its usual severity.

From 48 to 72 hours elapse from the chill or rigor to the first appearance of eruption. The temperature at this time, or very soon after the appearance of the eruption, drops to normal, and all the other symptoms improve correspondingly, leading the patient to believe that all trouble is over. In this he would be sadly mistaken if his disease were the smallpox of former epidemics, but as it prevails at present the initial stage constitutes, in very many cases, the principal part of the illness. The patient now frequently leaves his bed not to return to it again.

The eruption makes its appearance as minute papules, being first seen as a rule on some parts of the face, the forehead and the wrists.

FIG. 3.—Moderately severe form of smallpox in an unvaccinated adult, on eighth day of eruption. Semi-confluent on face, discrete on trunk and extremities.

Two or three days usually elapse before the outbreak is complete. The papules are sensibly elevated above the surface of the skin, and as they develop they assume the peculiar dense and firm character so commonly described. They change into vesicles somewhat earlier than usual. Not infrequently on the second or third day of the eruptive stage, distinct vesicles are seen. The peculiar condition known as umbilication may be seen in some of the lesions, but not in all. Frequently as early as the fourth or fifth day the vesicles change into pustules, and almost immediately shrinking and drying begin on the face, and a little later on other parts of the body. In some cases the eruption runs a course somewhat longer than that described, but in no instance have I seen it as long and tedious as in what might be styled normal smallpox. In the majority of cases the lesions are discrete and sparsely set. A few, however, exhibit the lesions more copiously, even to the extent of their assuming the semiconfluent or confluent form on the face, and sometimes on parts of the extremities also. Even in these cases the course of the eruption is abnormally short.

In the mildest cases the eruption, instead of passing imperfectly through the various phases of development common to the disease, assumes an abortive form, and recedes at a very early period; or else it develops rapidly into more or less dwarfed forms. A very common phase for the eruption to assume is for the papules to develop into solid conical elevations with small vesicles at their summit containing sero-purulent fluid. When dessication occurs, which is always rapid, and the thin crusts have fallen off, the solid part of the pock remains for a long time, giving the appearance of warty excrescences on the skin. This unsightly condition is most frequently seen on the face, but it eventually disappears without leaving any permanent disfigurement.

It is evident from the behavior of the eruption that the most striking peculiarity of this mild type of variola is the comparatively slight changes that occur in the skin. The lesions, instead of actively involving the deeper layers of the cutaneous integument, appear to develop between the outer epidermis and the layer of cells immediately covering the papilla and in the later suppurative changes the true skin becomes only mildly involved. Hence, dermatitis and the consequent intumescence, so common on the face and head in variola vera, are either absent or very mild, and the necrotic changes are, of course, greatly limited. The pustules, therefore, desiccate rapidly, forming comparatively thin scabs, which, when they have fallen off, leave pigmented spots, and but little or no pitting. Even in cases exhibiting a considerable degree of confluence on the face the eruption behaves in the same way. When such a case has reached the stage of pustulation a wonderful transformation of the features of the patient is often seen in the course of three or four days by the speedy subsidence of swelling and rapid shedding of the scabs.

In consequence, therefore, of the mild character and short course of the pustular stage, secondary or suppurative fever is by no means a prominent symptom. Indeed, it is not seen at all in the vast majority of cases, and in those in which it does occur it is moderate and of short duration, lasting only a day or two. Severe implication of the mucous membrane of the nasal cavities, the mouth, pharynx, and upper air passage, which during the pustular stage is often an accessory cause of secondary fever and of death, is not met with in the present type of variola. The phenomenal mildness of the symptoms as a whole, and especially during the suppurative stage when life is usually placed in greatest jeopardy, explains why the mortality from the disease in various parts of the United States for the last two or three years has been practically nil.

Those familiar with smallpox will recognize in the description I

FIG. 4.—Severe form of smallpox in an unvaccinated, on eighth day of eruption. Confluent on face, semi-confluent on extremities.

have given a clinical picture of mild varioloid; and yet it must be remembered that in nearly all of the cases which have come under my observation, and which I am describing, there was no known modifying influence operating, such as results from vaccination or a previous attack of the disease. Why smallpox in the unvaccinated should present itself so generally in the present exceptionally mild form is a question I shall not undertake to answer.

In view of the difficulty that has been experienced in recognizing the disease in its present type, I wish to say a few words on the diagnosis, more especially the differential diagnosis between it and some of the affections with which it has been more frequently confounded. Of these, varicella, impetigo contagiosa, and pustular syphiloderm especially claim consideration.

The onset of varicella is very different from that of variola. There is usually no distinct febrile stage preceding the eruption. Occasionally a rise of temperature precedes the cutaneous manifestations by a few hours, but far more frequently these two symptoms appear simultaneously. It is true, in many cases of extremely modified smallpox no reliable history of initial stage can be obtained, so that the diagnosis in such cases must be made from the appearance and behavior of the exanthem alone. It is important to bear in mind the following facts: That the lesions of varicella make their appearance as distinct vesicles containing perfectly clear serum; that they are usually seen first on parts of the body which are covered with clothing, and especially on the back, where they are apt to be most abundant; that they make their appearance in successive crops, and may be seen in every stage of development; that they vary very greatly in size; that they are unilocular, and have an epidermic covering so delicate as to be readily broken by the finger nail; that they are rather soft and velvety to the touch; that many of them enlarge to a coniderable circumference by peripheral extension, while others are as small as millet-seed; that they are not umbilicated, except by desiccation beginning in their centers; that they run their course to the formation of crust in two to four days; that the crusts are thin, brown and friable, and when they have fallen off red instead of pigmented spots remain; and that but few of the lesions are followed by permanent scars.

By way of contrast I would say that the exanthem of smallpox first appears in the form of papules, which are firm and dense to the touch, feeling somewhat like grains of sand buried in the skin; that they usually appear first on the face, and then on other parts of the body; that the papules slowly develop into vesicles with turbid or milky contents; that the vesicles in well marked cases are umbilicated; that they are multilocular, and have an epidermic covering so dense and firm as not to be easily broken by the finger nail; that the eruption prefers the exposed parts of the body, such as the face, hands and arms, being often only sparsely seen on the trunk; that the vesicles are usually quite uniform in size; that they change into pustules; that the eruption requires in severe cases twelve or more days to pass through its various stages, while in extremely mild cases not more than five or six days are required; that the crusts which form are thick and very dark, and when they have fallen off there remain pigmented spots and more or less pitting.

While each group of symptoms just enumerated is descriptive respectively of chickenpox and smallpox, and while there should be no difficulty in differentiating between these diseases in any case in which either group is complete, yet it must be admitted that

FIG. 5.—Showing rapid shedding of crusts between eighth and twelfth day.

smallpox sometimes occurs, as at present, in a form so atypical as to make it difficult to decide to which category the symptoms belong. It may, however, be stated in a general way that a mildly febrile eruption appearing without prodromal symptoms, being distinctly vesicular from the beginning and commencing to desiccate on the second or third day, should be regarded as chickenpox; and; on the other hand, an acute exanthem preceded by an initial stage of forty-eight hours, in which the temperature was distinctly elevated, beginning as papules and ending in vesicles or vesico-pustules, even though the period of evolution be short, should be regarded as smallpox. At any rate, it would be advisable for the safety of the public to regard such a case as suspicious, and surround it with such precautionary measures as are best calculated to prevent the spread of infection.

Impetigo contagiosa is an acute contagious disease of the skin rarely attended by rise of temperature. Unlike variola, it is not preceded by an initial stage, nor does it begin as papules, but appears at once in the form of vesico-pustules, which spring up on an apparently normal skin. They are quite superficial, and enlarge by peripheral extension, usually attaining the size of a silver dime. They remain very flat in comparison with the conical appearance of the vesicles of variola. The crusts which form may be either thin or thick, varying from a straw color to a greenish yellow or brownish hue. They are generally very friable, lightly adherent, and crumble off in small pieces. When the thicker crusts are forcibly removed a purulent surface is exposed, but no deep ulcer. After the natural shedding of the crusts there remain for a short time red spots, but never any scars.

The infecting principle resides in the vesico-pustules and is communicated by contact or accidental inoculation. When the disease appears in an individual new lesions may be carried by the finger nails to any part of the skin which is excoriated. Whether considered in part or as a whole, the nature and symptoms of impetigo contagiosa differ so widely from that of smallpox that it seems almost imposible for these diseases to be confounded, and yet one is sometimes mistaken for the other.

The lesions of pustular syphiloderm often resemble very closely those of smallpox, so that some care is necessary to distinguish between the two. The difficulty is increased from the fact that the eruption in either case is preceded by fever and various aches and pains, and that the lesions begin as papules and end as pustules. Instead of appearing all at once, the eruption usually comes out in successive crops. The papule progresses by the formation of a minute vesicle at its summit, and as this develops, its contents first become turbid and then pustular. Sometimes the pustule is limited to the apex of the papule, which later, in the meantime having enlarged considerably, gives to the pustule the apearance of resting upon a highly indurated base. At other times, or in different lesions of the same case, the entire papule may become involved in the suppurative process, which,when attended by a good deal of

FIG. 6.—Well marked case of chickenpox, on fifth day, showing lesions in various st ιges of development.

ulcerative action, is sure to be followed by deep and rather peculiar scars. When the ulceration is not excessive, the pustules dry up and form dirty-looking crusts of a brown or greenish-yellow color, and are quite friable. After the crusts have fallen off, the indurated base gradually disappears often, indeed, quite rapidly under treatment, leaving pigmented discolorations of a dark coppery hue.

Pustular syphiloderm may be distinguished from smallpox by the milder constitutional symptoms during the initial stage; by the absence of shot-like induration of the papules; by the formation of small vesicles at the summit of the papules; by the large, indurated base of each vesicle; by lesions appearing in successive crops; by the absence of umbilication; by tendency to ulceration of some of the lesions; by the comparatively thin, brown and friable scabs; by discolorations of a dark coppery hue after the scabs have fallen, and by concomitant symptoms of syphilis.

In considering the diagnosis of this affection much valuable information may often be gained by inquiring into the whole history of the case as well as carefully observing the course of the cutaneous lesions. To those accustomed to the appearance of smallpox there is something noticed in the general aspect of a syphilitic eruption, however similar to that of the former disease, which at once excites suspicion that it is not variolous. If to this can be added a history of syphilis, suspicion may be converted into certainty.

821 North Broad Street, Philadelphia.

## Figures and Tables

**Fig. 1. f1:**
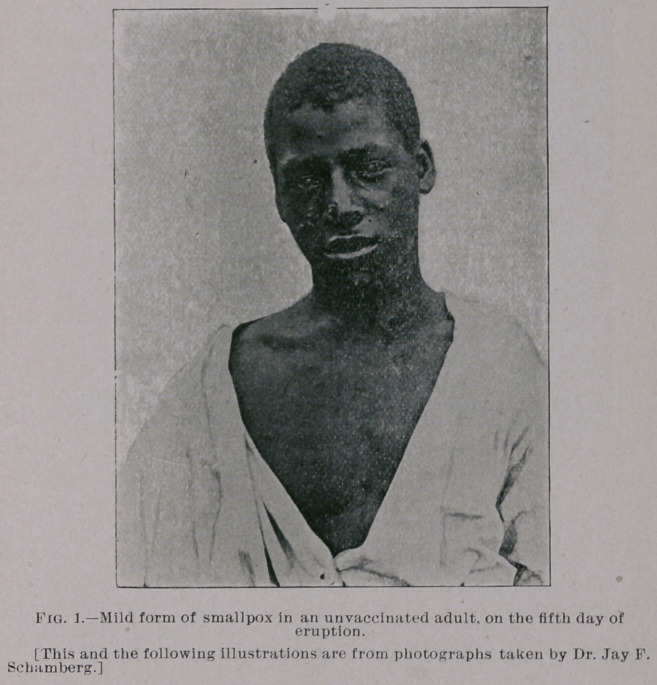


**Fig. 2. f2:**
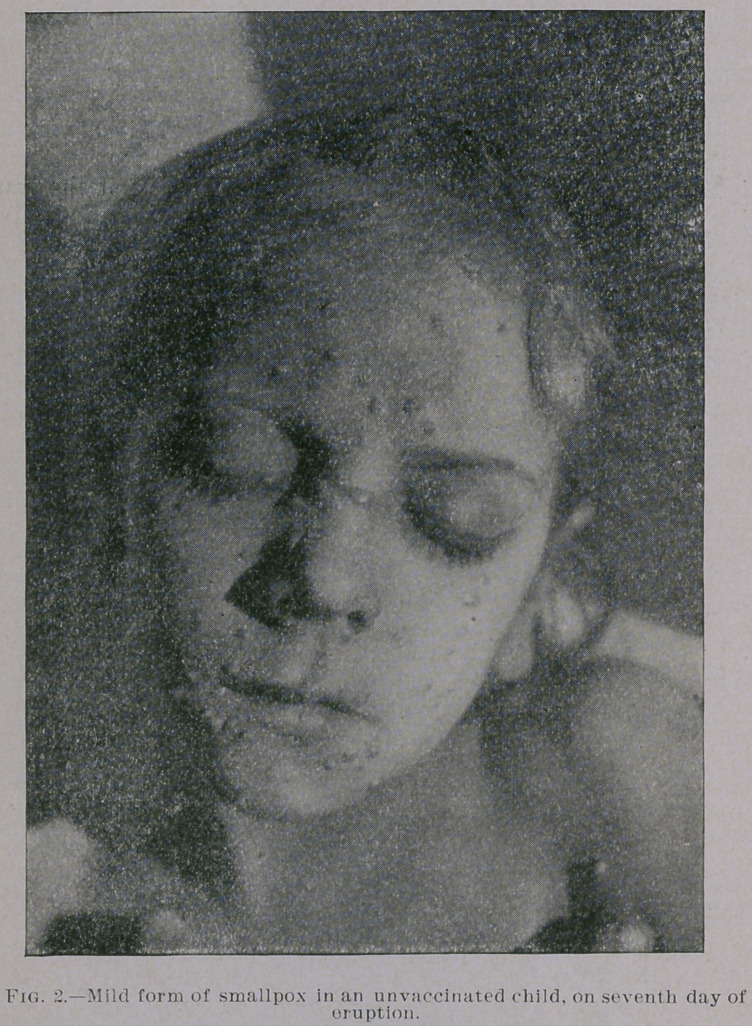


**Fig. 3. f3:**
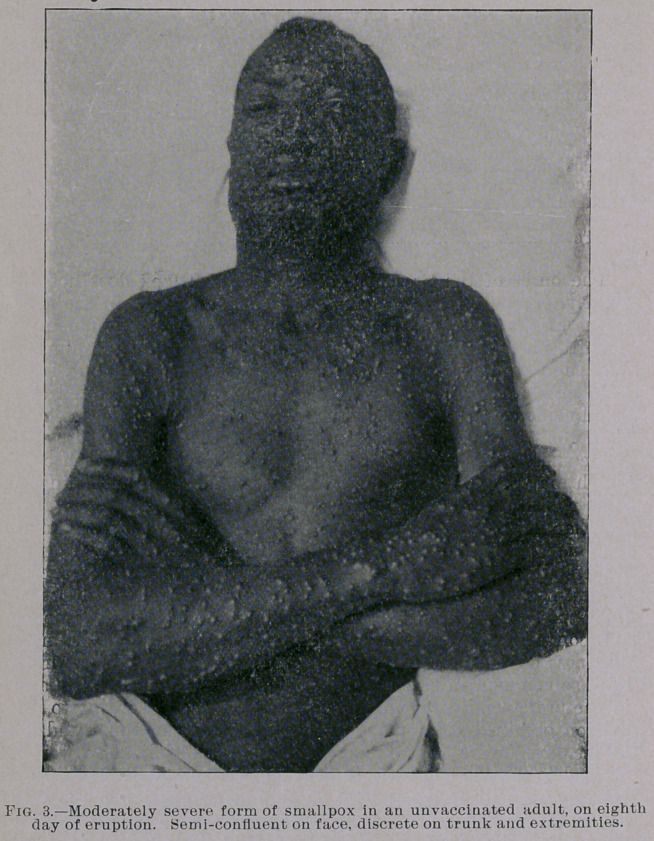


**Fig. 4. f4:**
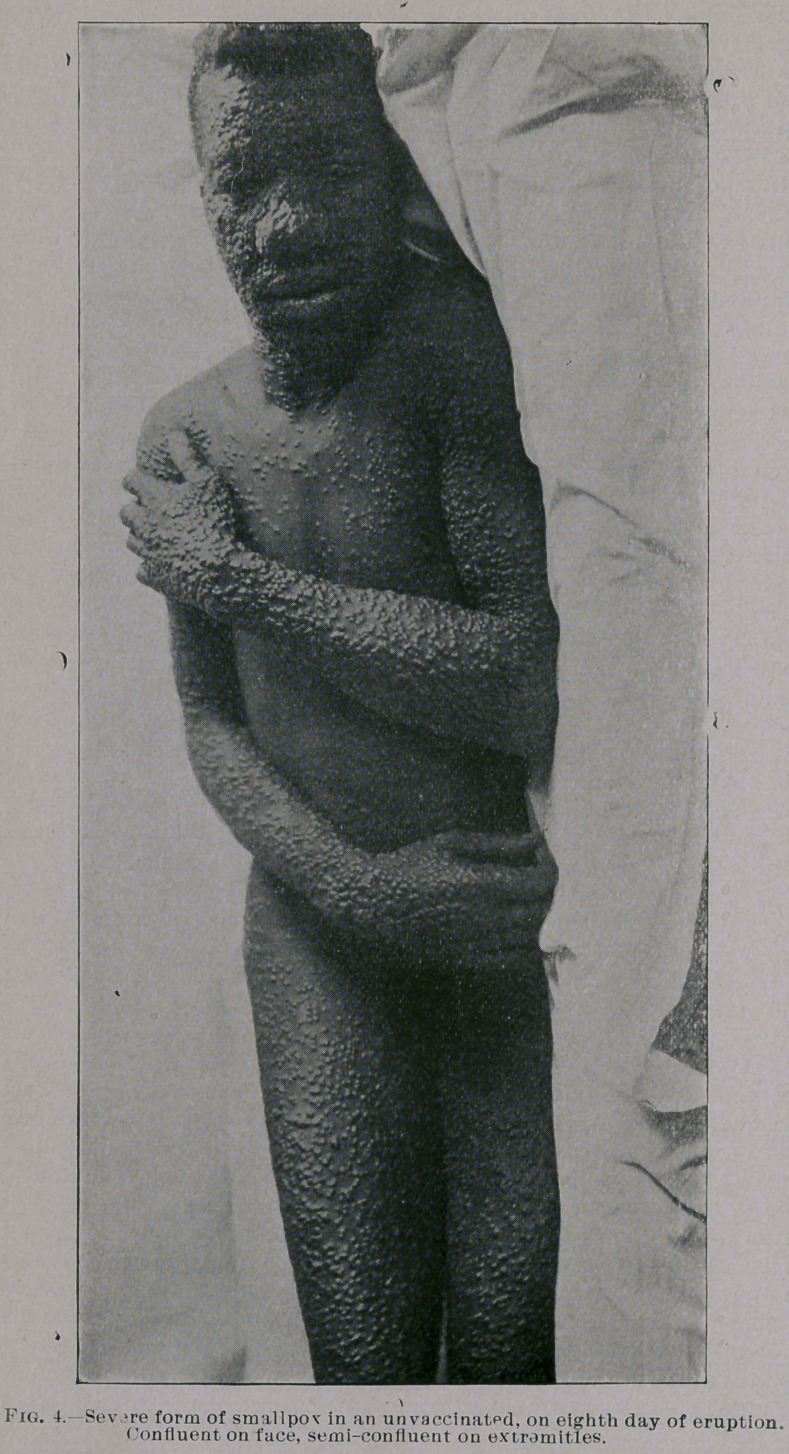


**Fig. 5. f5:**
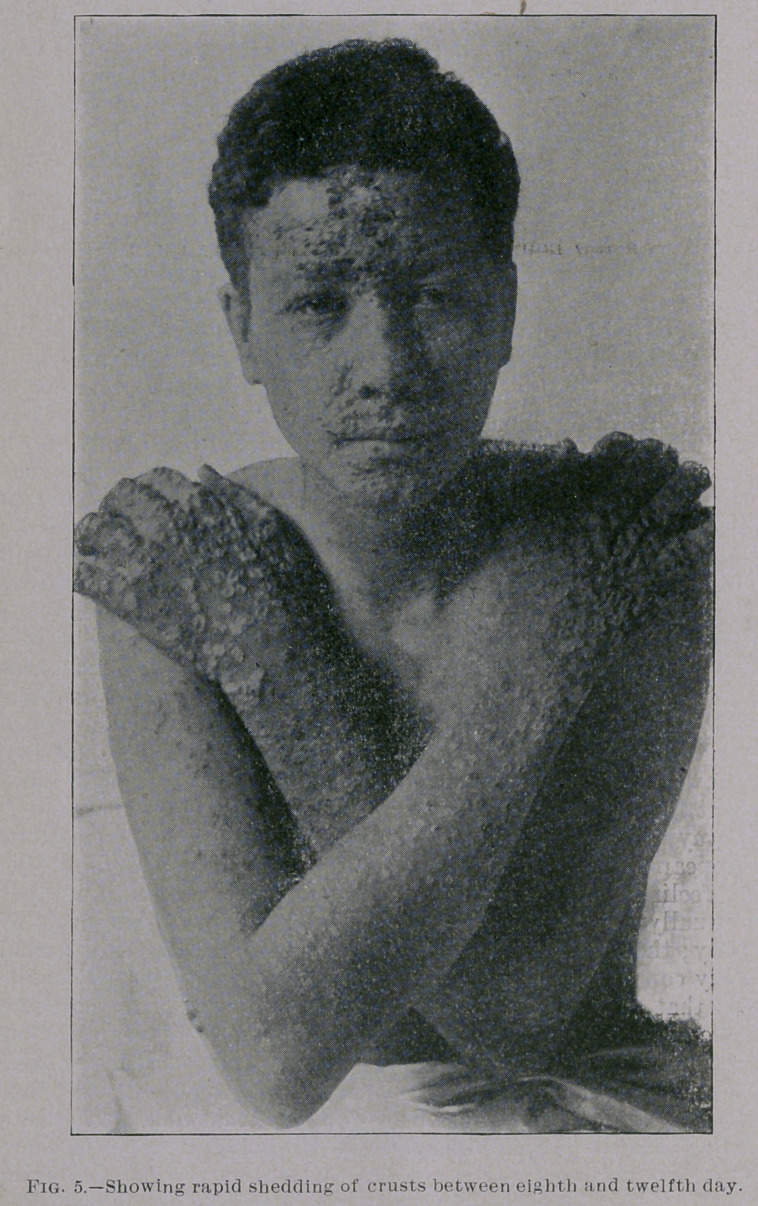


**Fig. 6. f6:**